# Alexithymia and Facial Mimicry in Response to Infant and Adult Affect-Expressive Faces

**DOI:** 10.3389/fpsyg.2021.635648

**Published:** 2021-08-04

**Authors:** Marc A. Nordmann, Ralf Schäfer, Tobias Müller, Matthias Franz

**Affiliations:** Medical Faculty, Clinical Institute for Psychosomatic Medicine and Psychotherapy, University Hospital of the Heinrich-Heine-University, Düsseldorf, Germany

**Keywords:** alexithymia, baby schema, affect regulation, EMG, facial mimicry

## Abstract

Facial mimicry is the automatic tendency to imitate facial expressions of emotions. Alexithymia is associated with a reduced facial mimicry ability to affect expressions of adults. There is evidence that the baby schema may influence this process. In this study it was tested experimentally whether facial mimicry of the alexithymic group (AG) is different from the control group (CG) in response to dynamic facial affect expressions of children and adults. A multi-method approach (20-point Toronto Alexithymia Scale and Toronto Structured Interview for Alexithymia) was used for assessing levels of alexithymia. From 3503 initial data sets, two groups of 38 high and low alexithymic individuals without relevant mental or physical diseases were matched regarding age, gender, and education. Facial mimicry was induced by presentation of naturalistic affect-expressive video sequences (fear, sadness, disgust, anger, and joy) taken from validated sets of faces from adults (Averaged Karolinska Directed Emotional Faces) and children (Picture-Set of Young Children’s Affective Facial Expressions). The videos started with a neutral face and reached maximum affect expression within 2 s. The responses of the groups were measured by facial electromyographic activity (fEMG) of corrugator supercilii and zygomaticus major muscles. Differences in fEMG response (4000 ms) were tested in a variance analytical model. There was one significant main effect for the factor emotion and four interaction effects for the factors group × age, muscle × age, muscle × emotion, and for the triple interaction muscle × age × emotion. The participants of AG showed a decreased fEMG activity in response to the presented faces of adults compared to the CG but not for the faces of children. The affect-expressive faces of children induced enhanced zygomatic and reduced corrugator muscle activity in both groups. Despite existing deficits in the facial mimicry of alexithymic persons, affect-expressive faces of children seem to trigger a stronger positive emotional involvement even in the AG.

## Introduction

The dimensional construct of alexithymia consist of four facets: (1) difficulty in identifying feelings and distinguishing between emotional feelings and the physical sensations of emotional arousal, (2) difficulty in finding words to describe feelings to other persons, (3) limited imaginative processes, and (4) an external oriented thinking style (Bagby et al., 2020). Alexithymic impairment of affect processing has been investigated in epidemiological (Franz et al., 2008), clinical, and psychophysiological studies (Taylor and Bagby, 2013). Such studies have found a limited ability for empathy in alexithymic individuals as well as an impaired emotional awareness, with conceivable adverse consequences for social interaction and health (Kealy et al., 2018). Due to their limited ability to perceive, differentiate, and express emotions, persons with high levels of alexithymia often suffer from psychological disorders like depression, anxiety, eating disorders (Lenzo et al., 2020), somatization, somatoform disorders (Lankes et al., 2020), and autism (Ryan et al., 2020).

An important basis for the regulation of social interaction as well as emotional contagion is affect-driven (i.e., automatically steered for each basic affect system) mirroring (Gergely and Watson, 1996), also known as facial mimicry (Fischer and Hess, 2017). For the development of differentiated emotional competences, the affect-driven mirroring of facial expressions between infant and mother is crucial for bonding (Tronick et al., 1978). Thereby the affective state of the infant is not only passively perceived by healthy attachment figures, but is also intuitively sympathized, involuntarily imitated, and thus shared with the infant (Franz and Schäfer, 2009). This process takes place in an affect modifying manner (Fonagy et al., 2018), whereby the infant is encouraged in its development not to regard the imitated affects as those of the reference person, but to perceive them as a reflection of its own. Such an imitation does not take place unchanged but is adapted by the mother according to the situation. Thus, negative affects of the child, such as anger or fear, are not imitated uncommented by the attachment figure, but are weakened and modulated by grief or comforting smiles (Fonagy et al., 2018) which can be understood as a “detoxification” function (Franz and Schäfer, 2009). Affective disorders could have an influence on this complex process. Parents with symptoms of depression were linked to more neutral affect during face-to-face interactions with their infant (Aktar et al., 2017).

However, facial mimicry promotes affiliation between individuals and therefore has a strong impact on the formation and preservation of social relationships (Drimalla et al., 2019). An individual’s facial mimicry facilitates affect recognition of other faces (Hess and Fischer, 2014). It is already known that verbal and non-verbal emotion recognition are impaired in alexithymia (Lane et al., 1996; Senior et al., 2020). Furthermore, in studies with autistic participants, impairments in facial emotion recognition can be better explained by the construct of alexithymia than by autism (Ola and Gullon-Scott, 2020; Cook et al., 2013). Alexithymia may represent an acquired unconscious avoidant strategy against unwanted emotions and lead to a suppression of facial emotion recognition (Panayiotou et al., 2015). Scarpazza et al. (2018) and Sonnby-Borgström (2009) showed that not only is emotion recognition impaired in alexithymia but also facial mimicry. Alexithymic persons showed a decreased emotional mimicry compared to a control group (CG) when confronted with static pictures of affect-expressive faces from adults. With respect to these results, it can be considered that dynamic stimuli evoke stronger facial mimicry compared to static portraits and better represent everyday social interaction (Rymarczyk et al., 2016).

In contrast to faces of adults, an infant or baby face has specific facial characteristics, which are summarized under the term baby schema or “Kindchenschema” (Lorenz, 1943). Faces with a strong expression of baby schema appear more attractive, sweeter, and lovable (Luo et al., 2011; Borgi et al., 2014). Healthy caregivers are influenced by these peculiarities and offer security for the offspring (Alley, 1983; Nittono et al., 2012). Affect-expressive faces of children could therefore promote phylogenetic bonding behavior (Grossmann and Grossmann, 1990). This principle of infantile facial features influences attachment affinity of adults and the child’s own affective development (Glocker et al., 2009). Improved speech development and the development of motor skills are linked to the emotional responsiveness of the mother (White-Traut et al., 2018). High alexithymia levels in parents can negatively influence the affective resonance with their children (Mensi et al., 2020).

Human infant faces are biologically highly relevant stimuli that generate attention and caring behavior as described above and are implicitly associated with positive emotions regardless of gender and parental status (Senese et al., 2013). If alexithymia is an acquired involuntary avoidant strategy toward unpleasant emotions, the baby schema could help to counteract this avoidance strategy and lead to more facial mimicry in response to affect-expressive faces even in the alexithymic group (AG). It is hypothesized that the participants of the AG show decreased affect-responsive facial electromyographic activity (fEMG; facial mimicry) compared to the CG after presentation of all affect-expressive faces of adults. Further, it is assumed – due to the impact and the evolutionary importance of the baby schema – that after presentation of affect-expressive faces of children, the facial mimicry of the AG does not differ from the CG.

## Materials and Methods

This study investigates facial mimicry of mentally healthy persons (no ICD-10 F-diagnosis of a psychic/psychiatric disorder) with high and low alexithymia, based on fEMG activity of the corrugator and zygomatic muscle in response to the presentation of video sequences with dynamically animated facial affect expressions of children and adults according to five basic affects (fear, sadness, disgust, anger, and joy). Specifically, to study the effects of alexithymia as a personality trait on facial mimicry, the influence of mental illness should be minimized.

### Psychometric Instruments

The Toronto Alexithymia Scale (TAS-20; Bagby et al., 1994) is a common questionnaire used to measure the level of a person’s ability to identify and describe emotions, and the tendency to minimize emotional experiences and focus outward attention. The instrument includes 20 items that are rated on a five-point Likert scale. The TAS-20 uses the cut-off scores: <51 = low alexithymia, >61 high alexithymia (Bagby and Taylor, 1997). For experimental studies it is recommended to use the 33rd percentile with a cut-off value of 45 and the 66th percentile with a cut-off value of 52 to ensure correct group classification (Franz et al., 2008).

The Toronto Structured Interview for Alexithymia (TSIA; Grabe et al., 2009) is an assessment instrument for the detection of alexithymic disorders of affect perception, processing, and regulation for clinical and scientific purposes. In each case the respondent is asked to give examples of corresponding situations from his or her life in all answers. A detailed coding catalog enables a three-stage assessment of alexithymia development per item. Only non-representative norm data are available and used as orientation. The intraclass correlation reliability estimates correspond to 0.90 (*p* < 0.01) and the reliability estimates to 0.88 (*p* < 0.01). A combined use of TAS and TSIA is suggested for an effective assessment of alexithymia (Montebarocci and Surcinelli, 2018).

The Patient Health Questionnaire (PHQ-9; Kroenke et al., 2001) is a nine-point module for depression from the PHQ. The PHQ-9 score ranges from 0 to 27 and each point can be rated from 0 (not at all), 1 (on a single day), 2 (more than half the days), 3 (almost every day). Major depression is diagnosed when 5 or more items are rated with a 2 and one of the items indicates depressed mood or anhedonia. The internal reliability was Cronbach’s α = 0.89 in a representative primary care study.

The Beck Depression Inventory (BDI-II; Hautzinger et al., 2006) is a 21-question multiple-choice self-report inventory. Cut-offs apply to the BDI-II, with 0–13 points indicating no or minimal depressive symptoms, 14–19 points mild, 20–28 points moderate, and 29–63 points severe depressive symptoms. The internal consistency of the BDI-II in clinical and non-clinical samples is in the range of 0.84 ≤ α ≤ 0.94. In a patient sample, the retest reliability in the one-week period was *r* = 0.93.

The Structured Clinical Interview for DSM-IV (SCID; Wittchen et al., 1997) is a structured interview created to make reliable psychiatric diagnoses in adults according to the Diagnostic and Statistical Manual, fourth edition (DSM-IV). The SCID has two parts: one for DSM-IV Axis I Disorders (SCID-I) and another for DSM-IV Axis II Personality Disorders (SCID-II). For reasons of test economy and content, only schizoid personality traits were recorded in this study for SCID-II, because schizoid traits led to study exclusion.

The short version of the Autism Spectrum Quotient (AQ-k; Freitag et al., 2007) consists of three factors: social interaction and spontaneity, imagination and creativity, and communication and reciprocity. The sensitivity analysis showed a cut-off value of 17. Internal consistency of the three factors was between 0.65 and 0.87.

The 20-item prosopagnosia index (PI20; Shah et al., 2015) is a self-report instrument assessing the presence of prosopagnosia traits. The 20 items describe face recognition experiences. Respondents indicate the extent on a five-point scale (strongly agree to strongly disagree). The cut-off scores are 65–74 for mild, 75–84 for moderate, and 85–100 for severe developmental prosopagnosia. The Cronbach’s α of 0.96 shows a high internal consistency of the 20 items.

### Recruitment

**FIGURE 1:**
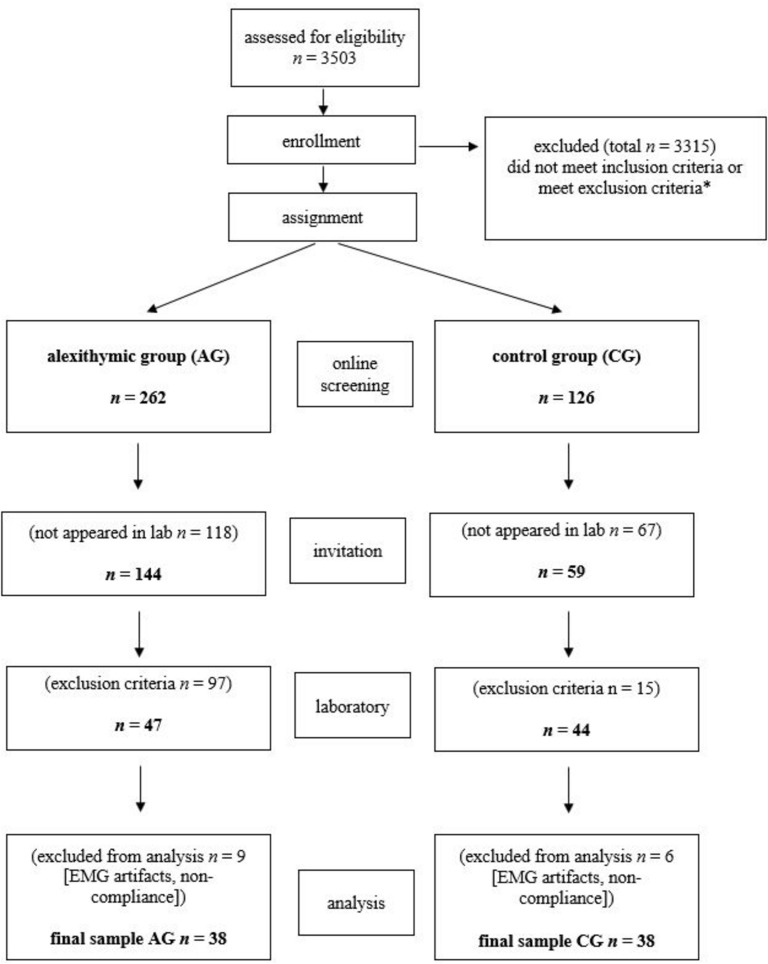
Flow of the participants for the given study procedures, assignment, and analysis. *Exclusion criteria were age <19 or >50 years, neurologic disease, particularly facial paresis, severe psychiatric disease, insufficient language skills; PHQ-9 sum-score < 9.

Volunteers were initially encouraged to participate in the study *via* posters and advertisements in social networks. After the study procedure had been fully described to the potential participants, written informed consent and data protection was obtained. Interested persons were directed to an online questionnaire (Leiner, 2014) and screened for sociodemographic variables (age, gender, siblings, and education), severe neurological or psychiatric diseases, particularly for existing current depressive burden by the German version of the PHQ-9, and the degree of alexithymia by the German version of the TAS-20. The online survey was completed 3503 times (see Figure 1). Exclusion criteria were: insufficient knowledge of German language; left-handedness; age below 18 or above 50 years; severe medical disorders, such as endocrine disorders or coronary heart disease; use of psychotropic medication; vigilance dysfunction; substance abuse; impairment of visus; neurological disorders (including neuropathy and botulinus-toxine usage); or psychiatric disorders [particularly psychosis, major depression (sum score < 14), anxiety disorders, addiction, eating disorders, schizoid personality disorder, and autism spectrum disorders]. In addition to the screening, the exclusion criteria were checked again in the laboratory at the beginning of the investigation by the structured interviews (SCID and TSIA), questionnaires (TAS-20, BDI-II, AQ-k, and PI20), and functional tests, to ensure a recruitment of mentally healthy probands in AG and CG. Finally, a rigorous “twin” matching or paralleling procedure with respect to sex, age, and educational level insured that both groups were very homogeneous and nearly matched with respect to sociodemographic characteristics. The finale sample AG (38) and CG (38) were built as mentioned (Franz et al., 2008) based on the TAS-20 sum score using cut-off scores <45 (threshold for the CG) and >52 (threshold for the alexithymic AG). The detailed selection process up to the final sample is shown in Figure 1.

### Sample Description

The total experimental sample included 76 participants (*n* = 38 each in AG and in CG) who met the inclusion and matching criteria. Table 1 shows the sociodemographic and psychometric properties of the study groups.

**TABLE 1:** Sociodemographic and psychometric characteristics of the sample, Welch df modification was applied for *t*-tests with unequal variances, for a sample size of less than 5 (education, children, and siblings) the exact Fisher test was used.

			AG			CG	*p*	Test parameters
	
Sociodemographic variables		*n*	%		*n*	%		
**Sex**								
Female		24	63		24	63		
Male		14	37		14	37	ns	χ^2^(74) = 2.63
**Education**								
GCSE		1	3		0	0		
GCE		28	74		26	68		
U/TC		9	23		12	32	ns	Fisher’s test = 1
**Children**								
Yes		2	5		1	3		
No		36	95		37	97	ns	Fisher’s test = 1
**Siblings**								
Yes		36	95		37	97		
No		2	5		1	3	ns	Fisher’s test = 1
**Age in years**								
	M (SD)			M (SD)				
	24.47 (5.35)	38		24.89 (5.43)	38		ns	*t*(74.00) = 0.34
**Psychometric variables**	M (SD)			M (SD)				
TAS-20	58.11 (4.58)	38		32.05 (5.56)	38		<0.001	*t*(74.00) = −22.31
TSIA	26.82 (6.51)	38		7.00 (3.48)	38		<0.001	*t*(56.53) = −16.54
BDI-II	7.74 (4.32)	38		1.89 (2.14)	38		<0.001	*t*(54.17) = −7.47
AQ-k	9.21 (4.32)	38		4.58 (2.40)	38		<0.001	*t*(57.87) = −5.87
PI20	40.03 (9.68)	38		34.68 (7.13)	38		<0.01	*t*(74.00) = 1.94

*ns, not significant; GCSE, General Certificate of Secondary Education; GCE, General Certificate of Education; U/TC, University/Technical College diploma; TAS-20, Toronto Alexithymia Scale-20 sum-score in laboratory setting; TSIA, Toronto Structured Interview for Alexithymia sum-score; BDI-II, Beck Depression Inventory II sum-score; AQ-k, Autism Spectrum Quotient – Short Version sum-score; PI20, Prosopagnosia Index sum-score. AG, alexithymic group; CG, control group.*

According to the criteria of the sampling procedure, groups differed with respect to alexithymia. The TAS-20 sum score was significantly increased in the AG compared to the CG [*t*(74) = −22.31, *p* < 0.001]. The clinical threshold criterion (Taylor et al., 1999) of >61 was met by *n* = 9. In addition, the TSIA confirmed the assignment of the participants to the AG in terms of a highly pronounced alexithymia [*t*(74) = −16.54, *p* < 0.001].

A clinical manifest depression of participants was prevented by recruiting only participants with a PHQ-9 sum score <9. The self-assessed depression measured by the BDI-II sum score showed a significant group difference, but all participants scored below the clinically relevant cut-off value of 13 [*t*(74) = −7.47, *p* < 0.001]. PI20, as a self-disclosure tool, showed a significant difference between AG and CG, but below the clinically relevant cut-off score of 65 for all participants [*t*(74) = −2.74, *p* = 0.008]. The AG also showed more pronounced autism typical characteristics – measured by AQ-k than the CG [*t*(74) = −5.78, *p* < 0.001]. However, none of the participants exceeded the clinically relevant cut-off value of 17.

The psychometric comparisons of AG and CG showed some significant group differences for these potentially confounding variables. However, the measured values for AG and CG were within the clinically unremarkable normal range. No defined clinical thresholds for the questionnaires (BDI-II, PHQ-9, PI20, and AQ-k) and the interviews (SCID) were exceeded, except the AG for TAS-20 and TSIA.

### Stimuli

The stimulus material consisted of video sequences with faces of adults and children expressing five basic affects (fear, sadness, disgust, anger, or joy). Each video starts with a neutral face and builds up a continuously more intense affect expression over two seconds to a maximum facial expression of affect (apex) which then is presented for one second. Original portraits of adults were taken from the picture set “Karolinska Directed Emotional Faces” (Lundqvist and Litton, 1998) and of children from the “Picture-Set of Young Children’s Affective Facial Expressions” (PSYCAFE; Müller et al., 2019). The most valid individual portraits per affect category (Goeleven et al., 2008) were used to create averaged and deindividualized affect-expressive portraits for each gender and affect (five basic affects and neutral expression). This was realized by a digital overlay of the individual faces and resulted in affect prototypical facial patterns of basic affects in a purified way. These averaged portraits served as material for the creation of the video sequences of each basic affect, gender, and age fading from a neutral to the maximum affect expression (2000 ms) by an interpolation algorithm (Abrosoft Fantamorph Deluxe 5). The resulting video clips consist of a dynamic sequence of naturalistic affect enrichment (2000 ms), followed by static presentation of the apex of every basic affect (1000 ms). Both the averaged portraits and the dynamic video sequences were created by means of the software Abrosoft Fantamorph Deluxe 5 using nearly 200 landmarks tagged to the most important face characteristics like nose, lips, and eyes. Validity aspects of the stimulus material (dynamic video stimuli) can be found in Müller et al. (2019). In this study, a specific mimic reaction could be measured for each basic affect displayed by both age groups.

### Experimental Procedure

**FIGURE 2:**
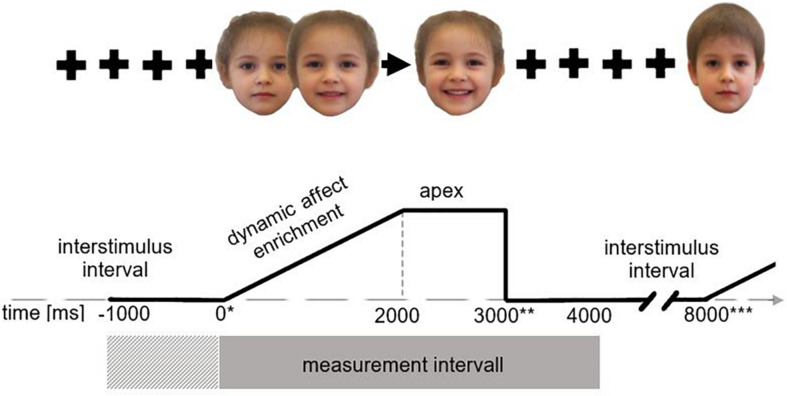
Presentation procedure. *During each trial cycle EMG activity was measured over 4000 ms (gray rectangle) from stimulus onset (indicated by*) with 1000 ms baseline correction (cross-striped rectangle), here shown for the example of a child’s face expressing joy. During the interstimulus interval (5000 ms) a fixation cross was presented from stimulus offset (indicated by **) to end of trial (indicated by ***); the trial begins with a neutral face which dynamically shows an increasing affect expression in the following 2000 ms. After 2000 ms the apex is reached and presented for 1000 ms.

The study was approved by the Ethics Commission at the Medical Faculty of the Heinrich Heine University Düsseldorf (registration number: 2016116024). All participants gave written informed consent prior to participation and received financial compensation. At the beginning of the experimental setting, the investigator introduced himself to the participants. After that, there was time to discuss the information sheet and to obtain the participants consent for the study. Before the experiment started, the visual perception, reactivity, and function of the facial nerve were tested by means of simple functional tests. This procedure was followed by participants’ completion of the psychometric instruments and the clinical interviews (TAS-20, BDI-II, SCID, TSIA, PI20, and AQ-k). This was done in a separate interview room. Only participants who showed no evidence of a mental disorder and whose results were below clinical cut-off values were included in the study. After that, the investigation cabin and all the apparatus were shown and explained to the participants. The participants were told in a general manner that “physical signals” will be measured during the video presentation of affect-expressive faces. Pictures and texts were presented on a 24-inch TFT-display (AMW) with resolution of 1920 × 1080 (60 Hz) set up in distance of 1 m. The experimental stimulus presentation and the trial coordination were controlled by using the software PsychoPy v1.82.01 (Peirce, 2007). Following the guidelines of Fridlund and Cacioppo (1986), the fEMG activity was measured bipolarly with Ag/AgCl miniature electrodes (Easy Cap E220N-CS-120) filled with electrode paste and attached to the left and right zygomatic and corrugator muscle regions. Two reference electrodes were attached to the mastoids, and two further electrodes were attached to the temporal head area measuring the electrooculogram (for offline artifact correction). The skin was cleaned with alcohol and rubbed with an abrasive electrode paste assuring impedances <10 kΩ (Fridlund and Cacioppo, 1986). After attaching the electrodes, the experiment started. First, participants were informed that they would see video sequences of facial affect expressions and that they should try to pay attention to every video and to empathize with the affect or emotion which is shown. Additionally, the instruction was given that the presented faces should not be actively imitated. The video clips which were used as stimuli were presented for 3000 ms as described above (2000 ms affect enrichment, 1000 ms apex presentation). Prior to every video clip a central black fixation cross on a white background appeared for 5000 ms as inter stimulus interval. The video clips were presented in randomized order. A total of 40 trials were shown (five emotions, two age groups, two genders, and two presentations). In addition to this, every participant was filmed during the whole procedure. This was to control participants’ cooperation to follow the instructions and participants’ compliance (especially involvement, attention, and vigilance instruction) during the experimental setting. Figure 2 shows the procedure of a trial exemplarily.

### Measurement of fEMG

During the stimulus presentation the fEMG signal was measured digitally using a sampling rate of 2000 Hz and a time constant of 0.016 s (digital polygraph EEG1100G; Nihon Kohden). The fEMG-signal was further processed offline with Brain Vision Analyzer. Moreover, a notch filter (50 Hz) was used to reduce electromagnetic noise and a high pass filter at 10 Hz and a low-pass filter was set at 1000 Hz. Two individual independent raters controlled the fEMG measurement for artifacts (e.g., motoric movements, electrode movements, or current voltage drifts). After this, the fEMG-signal was rectified and stepwise integrated over 4000 ms. Because of the use of dynamic affect enrichment within the first 2 s of the stimulus material and the expected deferred facial reaction, 4000 ms were selected for later analysis. This measurement section represents the fEMG data of the first 4 s (3 s stimulus presentation, 1 s interstimulus interval) after stimulus onset. The facial motoric activity was determined as the difference (μV) from the baseline activity being defined as the mean activity during the last 1000 ms before stimulus onset. This generated baseline-adjusted, integrated, and electromyographically measured muscle activity over the time of 0–4000 ms was used for the analysis of variance.

### Data Reduction and Statistical Analyses

The fEMG-data taken from both face sides (left and right) were averaged separately for each of the two different muscles: zygomaticus major (zygomaticus) and corrugator supercilii (corrugator). This was done for all participants and all stimulus presentation sequences. After that, every single trial (duration 4000 ms) was integrated which resulted in an accumulated overall fEMG activity parameter for muscle (zygomaticus and corrugator), emotion (fear, sadness, disgust, anger, and joy) and age (child and adult) of the stimuli. To rule out the possibility that group differences were not at least partly due to differences in depression (BDI sum score), autism (AQ-k sum score), and (impaired) face recognition (PI20 sum score), an analysis of covariance (ANCOVA) was performed including potential confounders, even though regarding these psychometric scales there were no clinically relevant differences between AG and CG. A four-factorial ANCOVA was conducted for the analysis with fEMG activity as dependent variable. If sphericity was violated, a Greenhouse–Geisser correction was conducted. For all sociodemographic and psychometric characteristics, non-parametric (Chi-square tests) or parametric test (*t*-tests) were used for analyses. Tests were performed two-sided by assuming an alpha error of *p*(α) = 0.05.

## Results

**FIGURE 3:**
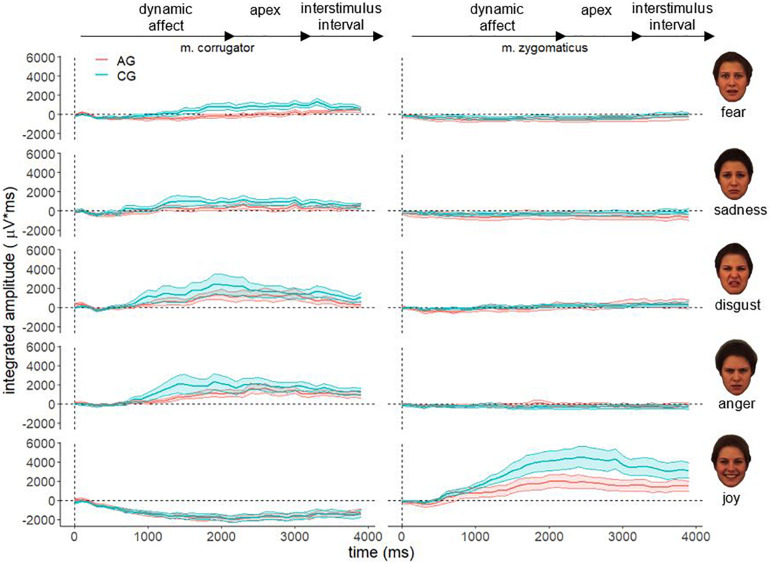
Electromyographical activity [μV integrated over 40 × 100 ms interval (μV × ms)] of corrugator supercilii (m. corrugator) and zygomaticus major [m. zygomaticus) for AG (red line) and CG (blue line)] in response to video clips of affect expressing faces of adults for all basic affects. Measurement period 0–4000 ms, vertical dotted line represents stimulus onset; ribbons represent the standard error.

Figure 3 shows the descriptive course of fEMG activity in both groups separately for muscle and affect over 4000 ms for adult faces. In general, the fEMG activity of the study groups is congruent with the dynamic affect enrichment of the stimuli, beginning from the neutral face to the apex. Approximately 500 ms after stimulus onset, parallel to the increasing facial expression of affect, an increasing fEMG activity that reaches its maximum after 2000 ms, corresponding to the apex in the videos, could be observed. Aversive stimuli (fear, sadness, disgust, and anger) resulted in increased fEMG activity in the corrugator. After presentation of the hedonic affect (joy), no – or a negative – fEMG response of the corrugator occurred. In contrast, presentation of joy resulted in very strong fEMG activity in the zygomatic muscle in both groups, whereas no fEMG response in the corrugator was detected for this stimulus. This differential activation of both muscles is consistent with the facial muscles involved in these different affect expressions. However, there are clear differences in the course of fEMG activity between AG and CG until the end of the stimulus presentation (3000 ms). These differences are also observed in the inter-stimulus interval until the end of the measurement period (4000 ms). In this last part of the measurement interval, after the end of the stimulus presentation (fixation cross), there is, as expected, a decrease in fEMG activity in both groups in both muscles studied across all affects.

**FIGURE 4:**
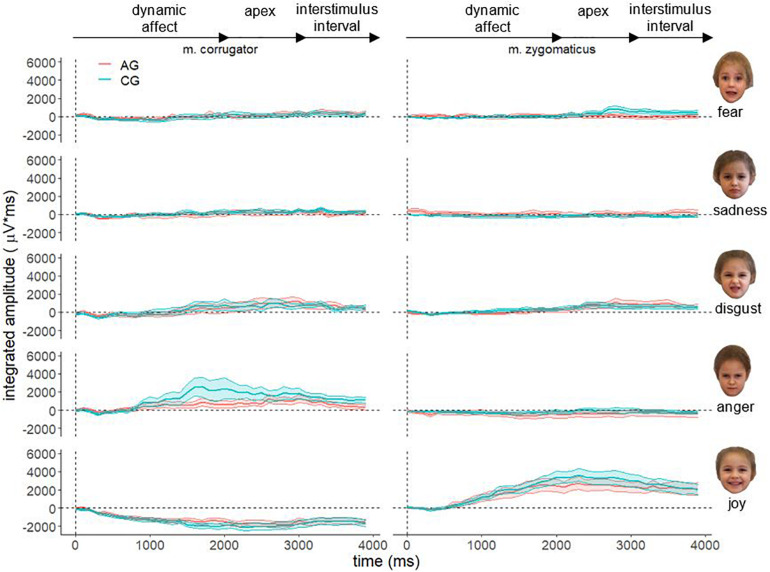
Electromyographical activity [μV integrated over 40 × 100 ms interval (μV × ms)] of corrugator supercilii (m. corrugator) and zygomaticus major (m. zygomaticus) for AG (red line) and CG (blue line) in response to video clips of affect expressing faces of adults for all basic affects. Measurement period 0–4000 ms, vertical dotted line represents stimulus onset; ribbons represent the standard error.

Figure 4 shows the descriptive course of fEMG activity in both groups separately for muscle and affect over 4000 ms for faces of children. As with faces of adults, fEMG response to faces of children are generally congruent with the dynamic affect enrichment of the stimuli. For the aversive stimuli (fear and sadness), a decreased activation is visible in both muscles for AG and CG. The affect disgust seems to evoke increased fEMG activity in the corrugator as well as in the zygomaticus muscle. The affect anger is associated with increased corrugator activity. After presentation of the hedonic affect (joy), strong fEMG activity emerged in the zygomaticus in both groups. However, the overall differences in the course of fEMG activity between AG and CG were lower for the faces of children than for the faces of adults.

Furthermore, all aversive stimuli (fear, sadness, disgust and anger) evoke an increased fEMG activity in the corrugator. However, after presentation of the hedonic effect (joy) no – or a negative – fEMG reaction of the corrugator occurred. In contrast, the presentation of joy in both groups led to a very increased fEMG activity in the zygomatic muscle, whereas no fEMG reaction was detected for this stimulus in the corrugator.

A four-way ANCOVA (within-subject factor emotion, muscle, and age; between-subject factor group; dependent variable fEMG; Table 2) including the covariates BDI sum-score, AQ-k sum-score, and PI20 sum-score revealed the following significant effects. There were two significant main effects for the factors group *F*(1,74) = 6.17, *p* = 0.015, η^2^ = 0.08 and emotion *F*(2.71,200.69) = 3.57, *p* = 0.018, η^2^ = 0.09. Four significant interaction effects for the factors group × age *F*(1,74) = 7.56, *p* = 0.008, η^2^ = 0.09, muscle × age *F*(1,74) = 15.58, *p* < 0.000, η^2^ = 0.17, muscle × emotion *F*(1.69,125.27) = 57.02, *p* < 0.000, η^2^ = 0.44 and for the threefold interaction muscle × age × emotion *F*(3.57,263.85) = 2.55, *p* = 0.046, η^2^ = 0.03. All remaining factors and interactions were not significant. Without including the covariates BDI sum score, AQ-k sum score, and PI20 sum score, the emotion × muscle interaction remains significant *F*(1.69,125.27) = 57.02, *p* < 0.001, η^2^*_*p*_* = 0.44.

**TABLE 2:** Analysis of covariance; dependent variable: integrated electromyographic activity (μV × ms) over the entire measured period (0–4000 ms) of the muscle: corrugator supercilii and zygomaticus major in response to the stimuli (videos with increasingly affect-expressive faces); between-subject factor: group (AG and CG), within-subject factor: emotion (fear, sadness, disgust, anger, and joy) and age (child and adult) with the covariates (AQ-k sum-score, PI20 sum-score, and BDI sum-score).

Factor	*df*_*Num*_	*df*_*Den*_	Epsilon	*F*	*p*	η^2^_*p*_
Group	1.00	74.00		6.17	**0.015**	0.08
Muscle	1.00	74.00		3.09	0.083	0.04
Age	1.00	74.00		0.13	0.718	0.00
Group × muscle	1.00	74.00		0.01	0.929	0.00
Group × age	1.00	74.00		7.56	**0.008**	0.09
Muscle × age	1.00	74.00		15.58	**0.000**	0.17
Group × muscle × age	1.00	74.00		0.00	0.946	0.00
Emotion	2.71	200.69	0.68	3.57	**0.018**	0.05
Group × emotion	2.71	200.69	0.68	0.31	0.798	0.00
Muscle × emotion	1.69	125.27	0.42	57.02	**0.000**	0.44
Emotion × age	3.36	248.44	0.84	1.14	0.335	0.02
Group × muscle × emotion	1.69	125.27	0.42	1.71	0.190	0.01
Group × emotion × age	3.36	248.44	0.84	2.09	0.095	0.03
Muscle × emotion × age	3.57	263.85	0.89	2.55	**0.046**	0.03
Group × muscle × emotion × age	3.57	263.85	0.89	1.70	0.158	0.02

**df*_*Num*_ indicates degrees of freedom numerator. *df*_*Den*_ indicates degrees of freedom denominator. Epsilon indicates Greenhouse–Geisser multiplier for degrees of freedom, *p*-values, and degrees of freedom in the table incorporate this correction. η^2^p indicates partial eta-Squared.*

**FIGURE 5:**
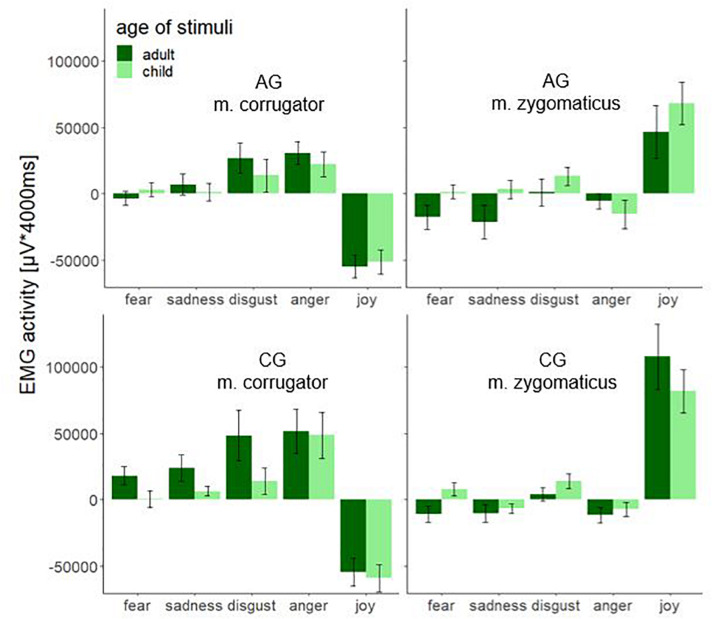
Electromyographic activity (μV × ms) over entire measurement interval (0–4000 ms) for the interaction effect consisting of the within-subject factors age (adult and child) and emotion (fear, sadness, disgust, anger, and joy) separated by muscle (m. corrugator and m. zygomaticus) and group (AG and CG). Negative EMG activity is caused by the baseline correction; error-bars represent the standard error.

Figure 5 shows the significant interaction effect age × muscle × emotion. The stimuli produced a significantly different fEMG response in the corrugator and zygomaticus muscles in AG and CG. Aversive affects (fear, sadness, disgust, and anger) produced a stronger response in the corrugator and the hedonic affect (joy) produced a stronger response in the zygomaticus muscle. This effect is important evidence that facial mimicry could be induced by the dynamic stimulus material. The AG as well as the CG showed a lower activity in the corrugator when aversive affects were demonstrated by faces of children. Zygomatic activity was increased in the AG except for anger for all affect-expressive faces of children compared to faces of adults. When comparing the groups, the largest differences were shown for faces of adults. The groups differed significantly less when presenting faces of children. In the AG the affect joy led to an increased zygomatic activity for faces of children, while the CG showed increased zygomatic activity for faces of adult. During the presentation of the affect joy, the groups differed very clearly for the faces of adults; there was no such clear group difference for the faces of children.

**FIGURE 6:**
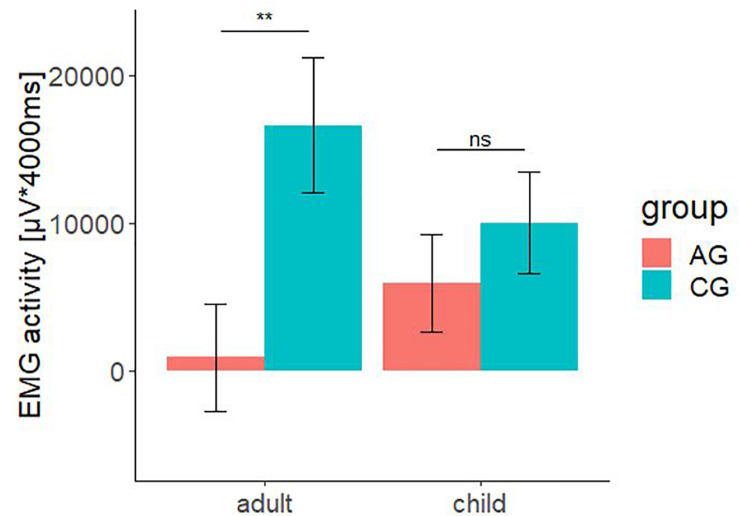
Electromyographic activity (μV × ms) over entire measurement interval (0–4000 ms) for the interaction effect consisting of the between-subject-factors group (AG and CG) and the within-subject factors age (children and adult faces). A significant lower EMG-activity was found for the AG in response to faces of adult. Negative EMG activity is caused by the baseline correction; error-bars represent the standard error. The symbol ** indicates *p* < 0.01. ns indicates not significant.

The significant interaction effect for the factors group × age is shown in Figure 6. The bar chart with standard error shows the integrated fEMG activity (μV × ms) over 4000 ms differentiated by type of stimulus (child versus adult) and the group factor (AG versus CG).

The total fEMG response (see Figure 6) over the zygomaticus and the corrugator muscles for five basic affects determined by the factors group and age as mean value with standard error for the AG were: adult: *M* = 919.08, SE = 3634.74; child: *M* = 5969.06, SE = 3282.44. For the CG, they were: adult: *M* = 16623.06, SE = 4559.81; child: *M* = 10041.54, SE = 3482.50. Bonferroni-adjusted *post hoc* analysis revealed a significant difference (*p* < 0.01) of the fEMG activity between the groups for the presented affect-expressive faces of adults but not for faces of children. The participants of AG showed a decreased fEMG activity in response to the presented dynamic affect-expressive videos of adults compared to the CG.

**FIGURE 7:**
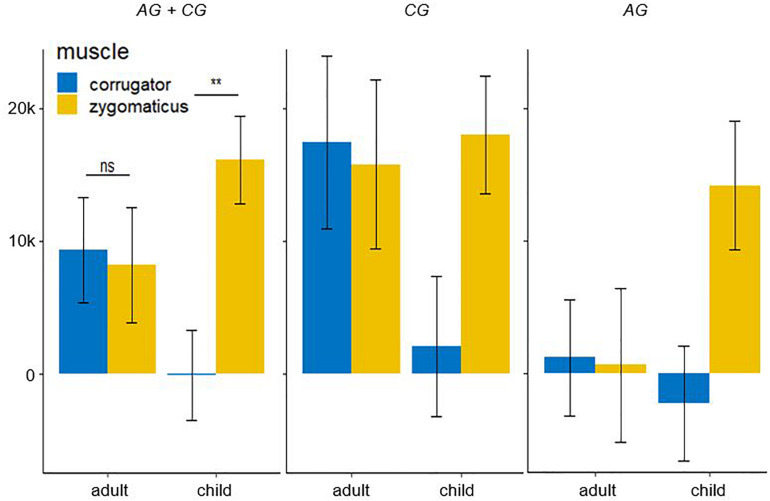
Electromyographic activity (μV × ms) over entire measurement interval (0–4000 ms) for the interaction effect consisting of the within-subject factors age (children and adult faces) and muscle (corrugator and zygomaticus) for both group. A significant lower EMG-activity was found for the corrugator in response to faces children. Negative EMG activity is caused by the baseline correction; error-bars represent the standard error. The symbol ** indicates *p* < 0.01. ns indicates not significant.

The fEMG response (see Figure 7; AG + CG) over 4000 ms for five basic affects determined by the factors muscle and age as mean value with standard error for corrugator were as follows: adult: *M* = 9341.47, SE = 3957.51; child: *M* = −94.71, SE = 3425.30. For the zygomaticus they were: adult: *M* = 8200.67, SE = 4320.29; child: *M* = 16105.32, SE = 3293.20. Bonferroni-adjusted *post hoc* analysis revealed a significant difference (*p* < 0.01) of the fEMG activity between the muscles for the presented affect-expressive faces of children but not of adults. The videos presenting affect-expressive faces of children resulted in a reduced fEMG activity of the corrugator muscle in both groups. The following diagrams differentiate this interaction effect regarding the two study groups. Due to the lack of significance in the threefold interaction group × muscle × age, no significance test was calculated for these comparisons. The fEMG response (see Figure 7; CG) of the CG over 4000 ms for five basic affects determined by the factors muscle and age as mean value with standard error for corrugator were: adult: *M* = 17460.19, SE = 6546.44; child: *M* = 2059.08, SE = 5305.65. For the zygomaticus they were: adult: *M* = 15785.93, SE = 6365.87; child: *M* = 18024.00, SE = 4451.43. With respect to the presentation of faces of adults, fEMG response of CG in both muscles was comparable, whereas affect-expressive faces of children resulted in decreased fEMG activity of the corrugator compared to the faces of adults. The fEMG response (see Figure 7; AG) of the AG over 4000 ms for five basic affects determined by the factors muscle and age was, as mean value with standard error for corrugator: adult: *M* = 1222.74, SE = 4388.62; child: *M* = −2248.51, SE = 4342.33. For the zygomaticus they were: adult: *M* = 615.41, SE = 5807.24; child: *M* = 14186.63, SE = 4862.23. The fEMG activity in response to the presentation of faces of adults was decreased compared to the faces of children. The only activation in the fEMG of the AG to all presented stimuli could be found in the zygomaticus muscle in response to the presented affect-expressive faces of children.

## Discussion

In this study, it was shown that facial mimicry could be induced in the AG as well as in the CG by the stimulus material. Muscle activity in the corrugator supercilii and zygomaticus major muscles reflected the valence of the presented affect (Dimberg et al., 2002; Müller et al., 2019), thus both groups showed increased muscle activation in the corrugator corresponding to aversive stimuli and increased muscle activation in the zygomaticus for stimuli with hedonic facial expressions.

By the significant interaction effect group × age within the ANCOVA a differential mimic response of the two groups related to the stimulus category age could be shown. For the faces of adults, the *post hoc* test revealed, as we hypothesized, that the precisely selected AG showed significantly decreased fEMG activity compared to the CG for all emotional conditions (Franz et al., 2021).

This group difference of the facial mimicry was not significant in the *post hoc* test for the affect-expressive faces of children. In contrast, the AG, like the CG, also displayed affect coherent facial muscle activation. This differential responsivity toward the facial affect expression of children may be seen as an effect of baby schema which facilitates facial mimicry also in the AG due to its evolutionary importance. Consistent with this, the probands of both groups showed an increased zygomatic activity compared to the corrugator muscle over all stimuli in response to the affect-expressive faces of children.

Based on the “detoxification hypothesis” a significant fourfold interaction muscle × emotion × age × group could have been expected. The fact that this interaction did not become significant could possibly be due to the low power caused by the limited sample size. In a reduced variance analytical model only with the corresponding muscles (joy – zygomaticus and anger – corrugator), the group × age × emotion interaction becomes significant *F*(1,74) = 4.54, *p* = 0.036, η^2^*p* = 0.06. Together with Figure 5, the directionality of the results becomes clear. The AG showed decreased facial mimicry to anger and joy during the presentation of adult faces. When presenting faces of children, there is no significant group difference in facial mimicry. Therefore, the reported age × group interaction must also be critically considered as it collapses the EMG activity across emotions and muscles. Considering that facial mimicry must have occurred (Figures 3, 4), we nevertheless interpret the group × age and muscle × age interaction as evidence of our hypothesis. For future studies, we recommend including more target muscles for aversive affects (for example, disgust – levator labii superioris; sadness – depressor anguli oris; fear – levator palpebrae superioris) (Ekman and Friesen, 1978) to maximize the probability of detecting the impact of facial mimicry.

In the study by Müller et al. (2019) it could already be shown that participants with generally low alexithymia showed more zygomaticus and less corrugator activity to the presented affect-expressive faces of children. Our results are generally comparable and point in the same direction. The AG showed almost no reaction of the corrugator, which is activated normally to express affects of anger, fear, sadness, and disgust. A fitting mimic mirroring reaction to the faces of adults like the CG showed by the zygomatic and corrugator activity could indicate a well-functioning affect perception and processing (Lundqvist and Dimberg, 1995). The observed reaction of the AG could be psychophysiological evidence for the known difficulties of alexithymic participants in affect perception and processing, especially for aversive affective states. Further studies demonstrated that alexithymic individuals show a decreased affective response, especially to emotional aversive stimuli (Schäfer et al., 2007; Starita et al., 2018). In a recent fEMG study with PTSD patients it could be shown that the extent of alexithymia predicts the recognition performance of negative affects (Passardi et al., 2019). Our results are in line with the mentioned studies with respect to faces of adults. The generally reduced facial mimicry to the predominantly emotional aversive valence of the depicted affects in the alexithymia group could be interpreted in terms of an acquired unconscious avoidance strategy against unwanted aversive affects (Panayiotou et al., 2015). From a neurobiological perspective, the automatic facial muscle activity of the perceiver of an affect generates neural feedback. This feedback triggers a similar affect in the perceiver. In turn, the perceiver may better understand the other person’s feelings (Barsalou et al., 2003). This process is explained by the so called facial-feedback hypothesis (Coles et al., 2019). The assumed alexithymic avoidance behavior could be limited to adult facial affect expressions. The mirroring facial expression of emotions toward children is probably more influenced by “hardwired” neurobiologically based processes because the perception and response to the phylogenetic attachment behavior of children directed to adult care givers (Grossmann and Grossmann, 1990) and enforced by the baby schema is too important for survival. So, this could be why emotional facial mimicry of adults in response to emotional faces of children is not affected even by the alexithymic impairment and affect avoiding.

Zhang et al. (2020) recently demonstrated that infants and toddlers communicate their needs and physiological states mainly through distinctive emotional signals, especially through the mimicking of the face which can provoke activation of special brain functions of mothers which are connected to reward and empathy (Hoekzema et al., 2017; Strathearn et al., 2009). Moreover, happy infant faces may also be experienced as rewarding for individuals without children of their own (Montoya et al., 2012). With this in mind, the fEMG response of the CG, which showed a decreased corrugator response and increased activation of the zygomaticus for the faces of children, can be considered as another validity aspect for the stimulus material.

Interestingly, a comparable reaction to the faces of children could also be observed in the AG. Human baby faces provoke caring behavior and positive reactions regardless of gender and parental status (Senese et al., 2013). Thus, individuals with a degree of alexithymia might also be encouraged to show an increased facial mimicry by the baby schema. Alexithymic participants, who generally show a reduced fEMG activity, displayed a rather strong positive affect expression when confronted with hedonic and aversive affective states of children. It is important to note that – due to the strict sample selection – these results are not contaminated by depression, other mental disorders, or schizoid personality traits.

People imitate emotional states depending on context, not just facial expressions (Hess and Fischer, 2013). This assumption is consistent with the results of this study, as children’s aversive affect expressions do not activate homologous mimic affect representations in the observer, but rather a basic caring program. Mirroring unmodified anger or fear to children, for example, would likely result in aversive disruption of the interaction. Also, alexithymic participants seem to be able to relate to this caring program. The reduced fEMG activity of the corrugator found in both groups during the presentation of affect-expressive faces of children would explain this aspect well. Thus, emotional mimicry also depends on contextual factors. The baby schema could be an important social contextual factor that promotes modified affect expression in the observer’s face rather than homologous facial mimicry in the sense of a protective and affect-regulating caregiving program. This lack of mimicry of aversive affective states within a phylogenetic caregiving program is also found in cooperative interactions (Hess and Bourgeois, 2010; Riehle et al., 2017). Negative emotions may be more strongly mimicked when the caring aspect is not as relevant, for example, when observers are confronted with unfamiliar adult faces. This fits with the results of our experimental study.

Despite careful testing and first validation aspects (Müller et al., 2019), the innovative dynamic stimulus material (PSYCAFE) may also have disadvantages in the expressiveness of affect compared to the already established stimulation material (Averaged Karolinska Directed Emotional Faces) with faces of adults. A validation study with encouraging results has already been conducted by our research group. However, it is also possible that differences in response to the faces of children or adults could be explained by the own-age bias (Anastasi and Rhodes, 2005), which implies that affect expressions are better recognized by individuals of the same age. Therefore, due to the lack of a CG of children, the extent to which this effect influences the results must remain open. Nevertheless, at least the combination of decreased activity of the corrugator muscle together with increased activity of zygomaticus in response to the emotional facial expression of children compared to expressions of adults could be interpreted as attenuated respective of the modified facial mimicry toward affect expressions of children in both groups (Fonagy et al., 2018). This could be understood as a mimic correlate of an interactional “detoxification” function of emotional aversive states, which is particularly important for the emotional development of children (Franz and Schäfer, 2009).

## Conclusion

To sum up, the high precision in the selection of a purified non-clinical sample and the challenging process of creating the stimulus material allows to investigate the effect of alexithymia on facial mimicry in response to the facial basic affects of children and adults. In this study, particular attention was paid to the facial expression, while alexithymic participants compared to non-alexithymic participants observed emotional faces of children and adults. A conclusion from the results could be that alexithymic probands are inhibited in facial mimicry to adult affect expressions and, in contrast, affect expressions of children can lead to hedonic facial mimicry despite increased alexithymia levels. Even alexithymic individuals can draw on complex affect programs in the sense of a “detoxification” effect, especially in interaction with children. Probably, these basal skills of alexithymic individuals could be used to promote and strengthen their emotional competencies.

## Data Availability Statement

The original contributions presented in the study are included in the article/supplementary material; further requests can be addressed to the corresponding author(s).

## Ethics Statement

The studies involving human participants were reviewed and approved by the Ethics Committee at the Medical Faculty of the Heinrich-Heine-University Düsseldorf. The patients/participants provided their written informed consent to participate in this study. Written informed consent was obtained from the individual(s), and minor(s)’ legal guardian/next of kin, for the publication of any potentially identifiable images or data included in this article.

## Author Contributions

MN, RS, TM, and MF contributed to the design and implementation of the research, to the analysis of the results, and to the writing of the manuscript. All authors contributed to the article and approved the submitted version.

## Conflict of Interest

The authors declare that the research was conducted in the absence of any commercial or financial relationships that could be construed as a potential conflict of interest.

## Publisher’s Note

All claims expressed in this article are solely those of the authors and do not necessarily represent those of their affiliated organizations, or those of the publisher, the editors and the reviewers. Any product that may be evaluated in this article, or claim that may be made by its manufacturer, is not guaranteed or endorsed by the publisher.
